# Treatment of enterocutaneous fistula with total parenteral feeding in combination with octreotide: a case report

**DOI:** 10.1186/1757-1626-2-177

**Published:** 2009-10-30

**Authors:** Enver Fekaj, Lulzim Salihu, Arbër Morina

**Affiliations:** 1 Department of Abdominal Surgery, University Clinical Centre of Kosova, Rrethi i Spitalit, str: p.n, Prishtina 10000, Republic of Kosovo; 2 University Clinical Centre of Kosova, Rrethi i Spitalit, str: p.n, Prishtina, 10000, Republic of Kosovo

## Abstract

**Introduction:**

The diagnosis and classification of fistulas based on anatomy, physiology and etiological criteria is the first important stage, conservative treatment consists on patient's stabilization. Finally, on complicated cases, when spontaneous closure fails, specific surgical approach should be applied.

**Case presentation:**

A 50 years-old women patient underwent four surgical interventions from the bowel gangrene, caused from the superior mesenteric vein thrombosis consequences. After fourth surgical intervention, at eighth post-operative day, the enterocutaneous fistula developed. On 20-th day, after enterocutaneous fistula developed, together with TPN, we administered also octreotide (100 micrograms/8 hours), for 48 hours. The reduction of fistula output, after treatment of TPN in combination with octreotide, compare the treatment only with TPN, was not significant (p < 0, 05). The enterocutaneous fistula, developed after fourth operation, has been spontaneously closed after four months.

**Conclusion:**

The fistula output, after treatment of TPN in combination with octreotide, compared with the treatment only with TPN, wasn't significant, in our case, (p < 0,05). We think that the optimum time for surgical treatment should not be based only on the period of time of conservative treatment, but other factors should be taken on consideration like: the pathology that has indicated the surgical treatment, the number of surgical interventions and period of time between these interventions.

## Introduction

In 75-85% of the cases the enterocutaneous fistulas appear because of consequence of iatrogenic agents like postoperative complications.

Approximately, in 15-25% of cases, gastrointestinal fistulas are spontaneous. Main causes are inflammatory bowel diseases, ischemic bowel disease, radiotherapy, pancreas malignancy etc. [1,2].

The diagnosing and classification of fistulas based on anatomy, physiology and etiological criteria is the first important stage. Conservative treatment consists on patient's stability. Finally, in complicated cases, when spontaneous closure fails, specific surgical treatment may be implemented [3].

The most important physiologic and determinant factor of fistulas is the daily output of intestinal fluid.

Conservative treatment consists on the control of electrolytic disturbance, nutritional support with enteral nutrition (EN), or parenteral and antibacterial therapy in the cases with systemic sepsis signs, or local inflammation accompanied with pain [4-7].

With the purpose to accelerate the spontaneous closure of fistulas, now, together with TPN, the somatostatin-14 or its synthetic analogue octreotide, can be given. In some studies, it has been concluded that TPN together with somatostatin-14, the closure time of fistulas can be reduced [8,9].

The reason that we are doing this presentation is to explain the effects of TPN, TPN in combination with octreotide, in management of enterocutaneous fistula of our case.

## Case presentation

A 50 years old Kosovan Albanian woman underwent four surgical interventions from the bowel gangrene, caused by the superior mesenteric vein thrombosis.

First surgical intervention resulted with resection of 150 cm of small bowel with T-T primary anastomosis.

Three months later, she underwent second surgical treatment. Intraoperationem, the small bowel (Ileum) gangrene has been ascertained about 35 cm, with free perforation and presence of fibro-purulent liquid in abdomen. The resection of ileum (about 50 cm) was realized and the ileostomy had been performed. The resection of distal ileum has been made about 8 cm from the ileocoecal valve and the remained part of ileum has been closed blindly.

Upon the second operation, on the seventh post-operative day, urgent surgical treatment was indicated. The patient was in a very difficult healthy condition; cardiac, renal and respiratory insufficiency with hypoalbuminemia emerged (18 g/l). Intra-operation the jejunum gangrene (5 cm), about 30 cm from Treitz ligament was ascertained. The resection of jejunum (10 cm) was performed with T-T primary anastomosis.

In ninth post-operative day, the enterocutaneous fistula developed. The fistula output was high (800-1300 ml/24 hours). This amount gradually went decreasing, and the fistula was closed spontaneously in thirteen days.

The patient was treated on Intensive Care Unit for 38 days.

After three months, the fourth surgical intervention was realized which consisted on re-establishment bowel continuity. The proximal part of small bowel was primary anastomosed with residual part of terminal ileum.

On the eighth post-operative day, the enterocutaneous fistula developed. The treatment started with TPN.

During the first month, the fistula output behaves from 200-900 ml/24 hours. On 20-th day after enterocutaneous fistula developed, together with TPN, we administered also octreotide (100 microgr./8 hours), for 48 hours.

The fistula output on 20-th day was 650 ml/24 hours, on 21-st day was 550 ml/24 hours, and on the 22-nd day, 520 ml/24 hours.

The reduction of fistula output, after treatment of TPN in combination with octreotide, comparing the treatment with TPN only, was not significant (p < 0.05).

Then, the octreotide administration stopped. The treatment continued with TPN only.

At the second month, the fistula output was between 85 - 690 ml/24 hours.

**Figure 1:**
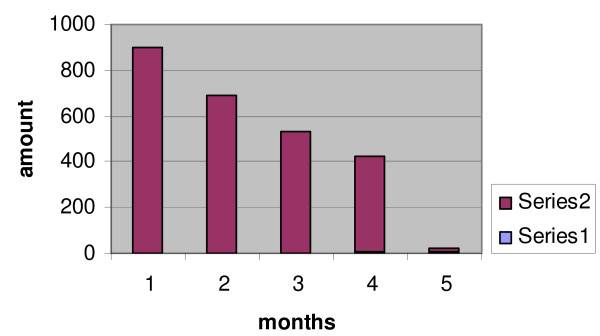
**Maximal fistula output throught months**.

At the third month, this amount reached up from 50-530 ml/24 hours, and at the fourth month this amount fluctuated from 15-420 ml/24 hours. At the beginning of fifth month, the fistula output was 2-15 ml/24 hours. The fistula output after four days decreased in 2 ml/24 hours. For two weeks this output remained constant (2 ml/24 hours), (see fig. 1)

After four months, the fistula has been closed spontaneously.

Now, two years have passed from the spontaneous fistula closure. Although, there was less than 80 cm residual small bowel, the patient has only 1-2 defecation/24 hours. She gained 14 kg.

The quality of her life is NORMAL.

## Discussion

Once the enterocutaneous fistula is diagnosed, conservative treatment should start immediately [10].

Since 1970, the essence of treatment in enterocutaneous fistula was an application of artificial nutrition in order to stabilize the patient, to induct the interruption of gastrointestinal activity and antibiotic administration in order to control the infection.

The application of TPN reduces the mortality rate and increases the level of spontaneous closure up to 60% of cases [5,11].

When the enterocutaneous fistula developed, TPN was applied. With the purpose to decrease the time of spontaneous closure of external gastrointestinal fistulas, lately, pharmacological agent treatment has been investigated.

A study has ascertained that somatostatin-14, in combination with TPN, decreases the time of spontaneous closure of fistula and significantly reduced the necessary time for treatment with TPN (13.9 +/- 1.84 days somatostatine-14 + TPN versus 20.4 +/- 2.98 days only with TPN, for (p < 0.05), and a consequent reduction in morbidity rate (35% somatostatin-14 + TPN versus 68, 85% only with TPN, for (p < 0.05), [8,9,12].

Two randomized studies, double blinded controlled and one blinded study crossover, has investigated the role of octreotide in gastrointestinal fistulas.

Only one of these studies ascertained the positive effect of fistula output reduction after 24 hours of treatment (53% reduction octreotide + TPN versus 9% TPN + placebo, n = 8 and n = 6, for p < 0.01).

Studies from Scott with C.A. and Sancho with C.A [13] didn't prove any significant effect, after treatment with octreotide in decreasing of fistula output.

The evidence suggests that if at the 1-st day, after somatostatin-14 treatment + TPN, the fistula output decreases for 50%, this presents an prognostic indicator for spontaneous closure of fistula.

On 20-th day we have applied octreotide (100 microgr./8 hours) + TPN for 48 hours.

In the first day, the output reduction was 15.4%, meantime in second day this decrease was only 5.5%. This reduction of the fistula output was not significant (p < 0,05). Then, we stopped treatment with octreotide. From the other authors it has been recommended to stop treatment with somatostatine-14 or octreotide, if, after 48 hours of treatment, there's no significant reduction of fistula output [14].

Enterocutaneous fistula in our case, after third operation, has been spontaneously closed after 13 days. The presence of ileostomy only 50 cm from the fistula has given its contribution. The enterocuteaneous fistula developed after fourth operation, has been spontaneously closed after four months.

In general, surgical treatment of enterocutaneous fistulas is indicated if spontaneous closure fails after 30-60 days of conservative treatment, although, in some cases, this treatment can be delayed for three months. But so, the surgical treatment is indicated if there is an obstruction distally of fistula [15].

In our case, we didn't indicate surgical treatment, because the patient had four surgical interventions. After these interventions, only 80 cm of small bowel remained. Any other intervention might cause a short bowel syndrome or may complicate with the development of enterocutaneous fistula.

## Conclusion

Although, in some investigations, it has been documented the positive effects of somatostatine-14 or octreotide by increasing of spontaneous fistula closure, in our case, where we have administered octreotide, but there wasn't significant decrease of fistula output after 48 hours of administration.

We think that: the optimum time for surgical treatment should not be based only on the long-time of conservative treatment, but, other factors should be taken on consideration, like:

1. The pathology that has indicated the surgical treatment,

2. The number of former surgical interventions and the timeframe between these interventions

The conservative treatment, though prolonged, it may lead to spontaneous closure of fistulas.

With all possible difficult complications in development, even it is prolonged, in specific cases, the conservative treatment should have higher advantages comparing with the surgical treatment of enterocutaneous fistulas.

## Comment

Changes on the study have been made in very careful manner without changing its essence. Since there were no macroscopic changes on the bowel for the chronic inflammatory disease, the histopathology examination has not been done. Thrombosis of vena mesenterica superior made gangrenous changes with segmental character in the small bowels (intestines). Enterocutaneous fistulae, after the third operation, has evolved because the patient was in severe condition with multi organ failure. While fistula after the fourth operation evolved because anastomosis of the small bowel was made 8 cm from ileocecal valve, a high risk place for performing the anastomosis. The purpose was to save the ileocecal valve and to save the important physiological function of terminal ileum.

## Competing interests

The authors declare that they have no competing interests.

## Authors' contributions

EF treated the patient and wrote the original paper.

LS treated the patient and edited the manuscript.

AM revised and edited the manuscript.

## Consent

Written informed consent was obtained from the patient for publication of this case report and accompanying images. A copy of the written consent is available for review by the Editor-in-Chief of this journal.
